# Engineering of many-body Majorana states in a topological insulator/s-wave superconductor heterostructure

**DOI:** 10.1038/s41598-017-02493-7

**Published:** 2017-06-14

**Authors:** Hsiang-Hsuan Hung, Jiansheng Wu, Kuei Sun, Ching-Kai Chiu

**Affiliations:** 1 Department of Physics, Southern University of Science and Technology of China, Shenzhen, Guangdong 518055 P.R. China; 2 Department of Physics, University of Texas at Austin, Austin, Texas 78712-1192 USA; 3 Department of Physics, The University of Texas at Dallas, Richardson, Texas 75080-3021 USA; 4 Condensed Matter Theory Center and Joint Quantum Institute and Maryland Q Station, Department of Physics, University of Maryland, College Park, MD 20742-4111 USA; 5 Department of Physics and Astronomy, University of British Columbia, Vancouver, BC V6T 1Z1 Canada; 6 Quantum Matter Institute, University of British Columbia, Vancouver, BC V6T 1Z4 Canada

## Abstract

We study a vortex chain in a thin film of a topological insulator with proximity-induced superconductivity—a promising platform to realize Majorana zero modes (MZMs)—by modeling it as a two-leg Majorana ladder. While each pair of MZMs hybridizes through vortex tunneling, we hereby show that MZMs can be stabilized on the ends of the ladder with the presence of tilted external magnetic field and four-Majorana interaction. Furthermore, a fruitful phase diagram is obtained by controlling the direction of magnetic field and the thickness of the sample. We reveal many-body Majorana states and interaction-induced topological phase transitions and also identify trivial-superconducting and commensurate/incommensurate charge-density-wave states in the phase diagram.

## Introduction

**Figure 1:**
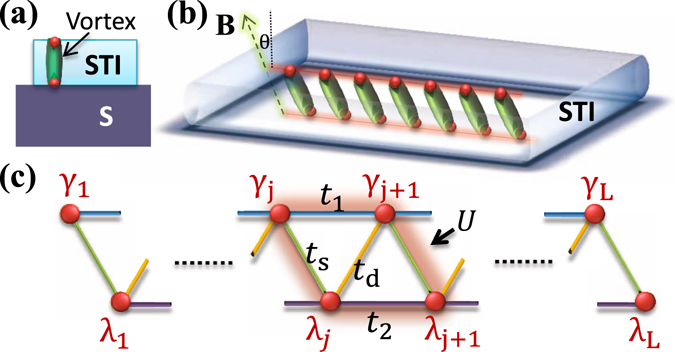
Experimental setup for a Majorana ladder. (**a**) Illustration of a pair of Majorana fermions (red dots) embedding respectively in the top and bottom of a vortex line (green tube) in a strong-topological-insulator thin film (STI, light-blue region) on an *s*-wave superconductor (S, dark-blue regions). (**b**) An array of vortices in the heterostructure, forming a two-leg Majorana ladder. An applied magnetic field *B* (dashed arrow) lines up vortices with angle *θ* and hence tilts the ladder system. (**c**) The tight-biding model for the Majorana ladder, describing top-chain (*γ*) and bottom-chain (*λ*) Majorana fermions coupled by intra-leg tunnelings *t*_1_ *γ*_*i*_ *γ*_*i*+1_ and *t*_2_ *λ*_*i*_ *λ*_*i*+1_, inter-leg tunnelings *t*_*s*_ *γ*_*j*_ *λ*_*j*_ and *t*_*d*_ *λ*_*j*_ *γ*_*j*+1_, and four-Majorana interaction *Uγ*_*j*_ *λ*_*j*_ *λ*_*j*+1_ *γ*_*j*+1_.

The exploration of various symmetry-protected topological states in quantum systems has become an intensively focused field in condensed-matter and AMO physics^1–3^. Quantum matter hosting Majorana zero mode (MZM), a particle being its own antiparticle, is of particular interest in the research forefront for its capability of revealing the intriguing nature of quantum entanglement and performing fault-tolerant quantum computation^4–12^. Recently, a pair of Majorana fermions in a one-dimensional (1D) system has been theoretically proposed and experimentally implemented in a semiconductor nanowire or a magnetic-atom chain on a superconducting substrate, producing an ideal quantum qubit^6, 10, 13^. However, efficient quantum information processing requires multiple qubits that can be practically manipulated. For this purpose, a more attractive candidate is the heterostructure of a three-dimension﻿al (3D) topological insulator (TI) film and an s-wave superconductor, which can carry a vortex array with a pair of MZMs embedding in each vortex and localizing around the top and bottom surfaces of the film, respectively^5^. The proximity effect of superconductivity has been confirmed that the superconductivity on the naked surface of the TI film is induced from the other side of the TI surface^14–17^, in contact with the superconductor as illustrated in Fig. 1(a). In experiments, MZMs can be observed only on the naked surface since the interface between TI and the superconductor has been buried. Currently, the observation of zero-bias conductance peak and spin selective Andreev reflection in the vortices shows the tentative evidence of MZMs^14, 18^.

Although the zero bias peak has been observed in the vortex cores of the naked TI surface, existence of the MZMs remains debatable in current heterostructure experiments due to two major issues. First, MZM and the low-energy Caroli-de-Gennes-Matricon mode^19^ (~0.01 meV) embedding in the vortex are indistinguishable due to the current energy resolution (~0.01 meV) in scanning tunneling spectroscope. Second, the TI should be thin enough such that the superconductivity can be proximity-induced on the naked TI surface^20^ but should be thick enough to suppress the Majorana hybridization on the top and bottom TI surfaces. In the recent experiment^14^, the thickness (~5 nm) of TI causes the order of 1 meV of the Majorana hybridization. By comparing with the superconducting gap (1 meV), this hybridization completely destroys MZMs. To save MZMs in this experimental setup, first we consider a 1D dense vortex array in the thin TI film and tune the chemical potential right at the Dirac point of the surface modes so that additional chiral symmetry is preserved. The symmetry suppresses the hybridization of MZMs on the same surface to zero. Hence, the interaction of four Majoranas becomes leading order^21–23^, so many-body Majorana wavefunctions have to be considered for the full characterization of the system’s quantum phases. Furthermore, when the vortex array is tilted by a magnetic field, the Majorana interaction assists a MZM to appear on the end of the vortex array. Such a many-body effect, though it was less investigated previously, not only provides additional degrees of freedom to engineer MZMs but also open an avenue to study interacting topological physics.

In this report, we propose a possible realization of a one-dimensional vortex array in a superconducting TI film device that can be represented by a tilted ladder model of many Majorana fermions associated with the Fu-Kane model^5^, as shown in Fig. 1(b). In this system, various intravotex and intervortex couplings between Majorana fermions are tunable with the control of the chemical potential and the vortex’s incline angle by an external magnetic field. Performing the density-matrix-renormalizaion-group (DMRG) calculations^24–27^, we obtain the many-body ground state of the system and present interacting phase diagrams as a function of these Majorana couplings. The presence of Majorana interaction enlarges the topological region of the Majorana ladder in the phase diagram; it leads to a MZM localized on the end of the ladder even in the presence of the Majorana hybridization.

## Results

### Experimental setup of a two-leg Majorana ladder

We start from the Fu-Kane heterostructure^5^, which is a 3D strong TI thin film on the top of an s-wave type-II superconductor. In this thin film, both top and bottom TI surfaces exhibit effective time-reversal-symmetric *p* ± *ip* superconductivity, via the superconducting proximity effect. Experimentally this setup has been demonstrated in Bi_2_Te_3_ thin films grown on a NbSe_2_ substrate^14, 17^, as shown in Fig. 1(a). With an external magnetic field turned on, vortices are generated on the TI surfaces and each end of the vortices hosts a MZM^28–30^. However, the induced superconducting gap on the naked (top) surface is much smaller than the bottom surface in contact with the superconductor^15, 17, 20^. Furthermore, the MZMs at the two ends of the vortex can tunnel through the vortex line and then hybridize, such that they do not possess zero energy. For this purpose, we consider a tilted magnetic field, which can effectively enlarge the distance between the MZM on the top and bottom surfaces, and weaken the hybridization.

Inspired by the one-dimensional vortex chain with a tilted magnetic field in the copper oxide thin films^31^, we consider a strongly anisotropic vortex array, which turns out to be a one-dimensional stripe along a certain direction determined by an external magnetic field, as shown in Fig. 1(b). With the tilted fields, the MZMs (red dots) at the top and bottom surfaces oppositely shift and form a tilted two-leg ladder, as shown in Fig. 1(c). On the same surface, the wavefunction of the MZM may overlap with its nearest neighbors and contribute to intra-leg hopping ${t}_{1}{\gamma }_{j}{\gamma }_{j+1}$ and ${t}_{2}{\lambda }_{j}{\lambda }_{j+1}$ for the top and bottom surfaces, respectively. The thickness of the TI determines the coupling between the top and bottom Majoranas along the same vortex line, ${t}_{s}{\gamma }_{j}{\lambda }_{j}$. As the magnetic field is titled, the hybridization between *γ*_*j*+1_ and *λ*_*j*_ becomes non-negligible, resulting in ${t}_{d}{\gamma }_{j+1}{\lambda }_{j}$. In addition to the single-particle hopping, there exists interaction among the MZMs. Assuming that the tilted angle *θ* is small enough such that the distance between *γ*_*j*_ and *λ*_*j*_ is less than that between *γ*_*j*+1_ and *λ*_*j*_, at the leading order we can have interaction stemming from four neighboring Majoranas in a closed loop ${\gamma }_{j}{\lambda }_{j}{\lambda }_{j+1}{\gamma }_{j+1}$. Thus, the whole Hamiltonian in the Majorana representation reads

1 $$\begin{array}{rcl}{\hat{H}}_{{\rm{M}}} & = & i\sum _{j=1}^{L-1}({t}_{1}{\gamma }_{j}{\gamma }_{j+1}+{t}_{2}{\lambda }_{j}{\lambda }_{j+1}+{t}_{d}{\gamma }_{j+1}{\lambda }_{j})\\  &  & +i{t}_{s}\sum _{j=1}^{L}{\gamma }_{j}{\lambda }_{j}+U\sum _{j=1}^{L-1}{\gamma }_{j}{\lambda }_{j}{\lambda }_{j+1}{\gamma }_{j+1},\end{array}$$

where *L* − 1 in the first summation indicates the open boundary condition. One feasible way to control *t*_1,2_ is to adjust the spatial distance between vortices, which can be artificially tuned via the magnitudes of magnetic fields; at the same time, however, the four-Majorana interaction is weaken. To keep the interaction strength, one needs to tune the chemical potential at the surface Dirac point to preserve additional chiral symmetry. The Majorana hybridization on the surface, which is forbidden by the symmetry, vanishes, and the Majorana interaction, which preserves the symmetry, survives^21–23^.

On the other hand, in the noninteracting limit, *U* = 0, the topology of the Majorana ladder is determined by *t*_*d*_/*t*_*s*_. The topological region where MZM reside on the vortex cores can be exactly determined at $|{t}_{d}/{t}_{s}| > 1$ (See **Method: Noninteracting Majorana ladder**). To solve the finite-*U* cases, one can transform the Majorana Hamiltonian Eq. (1) in terms of conventional fermionic operators ${\gamma }_{j}={c}_{j}+{c}_{j}^{\dagger }$ and ${\lambda }_{j}=i({c}_{j}-{c}_{j}^{\dagger })$ as

2 $$\begin{array}{rcl}{\hat{H}}_{{\rm{F}}} & = & \sum _{j=1}^{L-1}(\bar{t}{c}_{j}^{\dagger }{c}_{j+1}+{\rm{\Delta }}{c}_{j}^{\dagger }{c}_{j+1}^{\dagger }+h\mathrm{.}c\mathrm{.)}\\  &  & -\mathrm{(2}{t}_{s}+4U)\sum _{j=1}^{L}{n}_{j}+2U({n}_{1}+{n}_{L})+4U\sum _{j=1}^{L-1}{n}_{j}{n}_{j+1},\end{array}$$

where ${n}_{j}={c}_{j}^{\dagger }{c}_{j},\overline{t}=-{t}_{d}+i({t}_{1}+{t}_{2}),{\rm{and}}\,{\rm{\Delta }}=-{t}_{d}+i({t}_{1}-{t}_{2})$. Here we drop the constant energy shift after the basis transformation. We also consider a grand canonical ensemble such that the filling of fermions changes with the on-site term $-(2{t}_{s}+4U){\sum }_{j}{n}_{j}$.

The spinless fermionic Hamiltonian (2) has a similar structure to an interacting Kitaev chain^10, 32, 33^. However, we should emphasize that the realistic system (a Majorana ladder in a vortex chain) described by our model is fundamentally different from that in the previous study. Our proposed heterostructure provides a very different mechanism of tuning the model parameters, enabling the exploration of a wider phase diagram. For example, the last term, which has the form of nearest-neighbor electronic interaction, is actually determined by the overlap between four Majorana fermions in a plaquette ${\gamma }_{j}{\lambda }_{j}{\lambda }_{j+1}{\gamma }_{j+1}$. Therefore the strength *U* is related to the sample thickness and distance between two vortices on the same surfaces and can hence be fine tuned (compared with the hardly tunable electronic interaction in solid). To capture the salient physics, below we consider non-negative tight-binding parameters and interaction strength, i.e. ${t}_{1,2,s,d}\ge 0$ and *U* ≥ 0, the same intra-leg tunnelings on the top and bottom surfaces *t*_1_ = *t*_2_, and the inter-leg hopping *t*_*s*_ = 1 as the energy unit. Moreover, we are interested in the phases of the entire ladder, which should not be sensitive to the boundary condition, so we neglect the boundary term $2U({n}_{1}+{n}_{L})$ in Eq. (2) in following calculations.

Before getting into the details of the system’s phase diagram, we briefly point out that the original Hamiltonian of Eq. (1) has another equivalent form in terms of Pauli matrices *σ*_*x*, *y*, *z*_ as ${\widehat{H}}_{{\rm{S}}}={\sum }_{j}(-{t}_{s}{{\sigma }}_{j}^{x}-{t}_{1}{{\sigma }}_{j}^{y}{{\sigma }}_{j+1}^{z}+{t}_{2}{{\sigma }}_{j}^{z}{{\sigma }}_{j+1}^{y}+{t}_{d}{{\sigma }}_{j}^{z}{{\sigma }}_{j+1}^{z}+U{{\sigma }}_{j}^{x}{{\sigma }}_{j+1}^{x})$, which can be obtained through a Jordan-Wigner transformation ${\gamma }_{j}=({\prod }_{k}^{j-1}{\sigma }_{k}^{x}){\sigma }_{j}^{z}\,{\rm{and}}\,{\lambda }_{j}=-({\prod }_{k}^{j-1}{{\sigma }}_{k}^{x}){{\sigma }}_{j}^{y}$. This Hamiltonian describes a spin chain with transverse Zeeman field (*t*_*s*_), anisotropic Dzyaloshinskii-Moriya interaction (*t*_1, 2_)^34, 35^, and anisotropic exchange interaction (*t*_*d*_ and *U*). Our proposed heterostructure may thus find applications as a test bed to such interesting spin systems.

### Phase diagram

**Figure 2:**
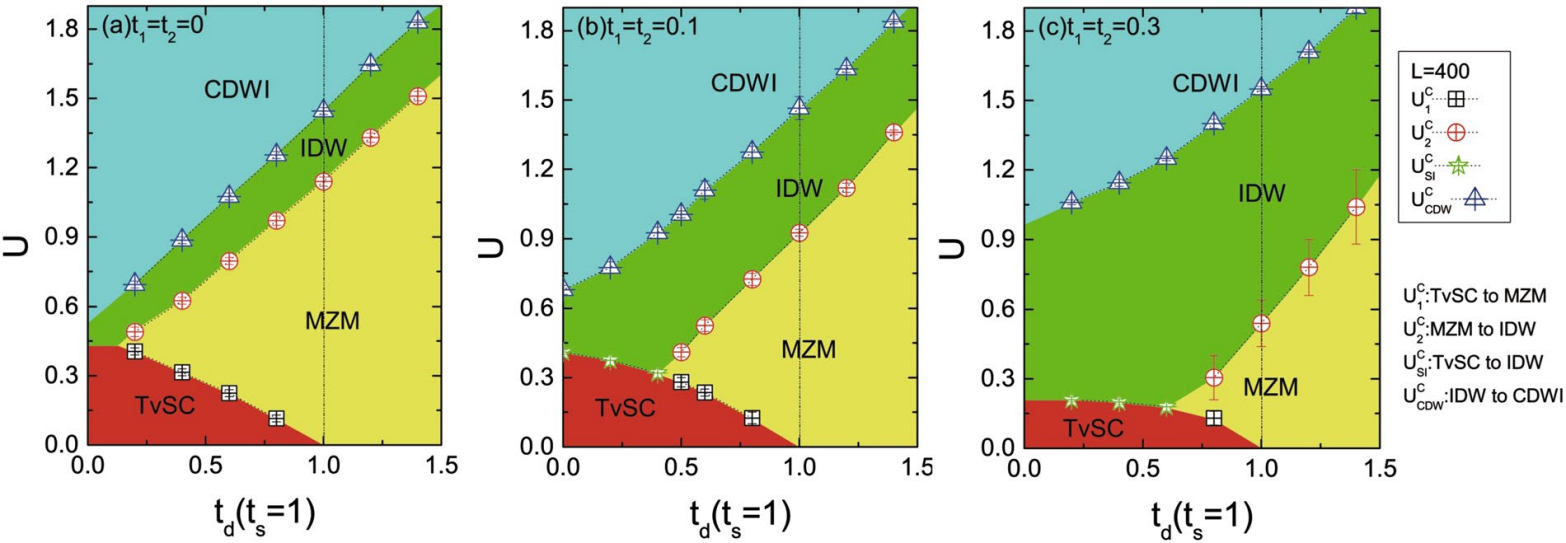
Phase diagram for various *t*_1,2_ values. The *t*_*d*_ vs *U* phase diagram of the interacting Majorana ladder at (**a**) *t*_1,2_ = 0 (**b**) *t*_1,2_ = 0.1*t*_*s*_ and (**c**) *t*_1,2_ = 0.3*t*_*s*_. The dot line locates the noninteracting topological phase boundary *t*_*d*_ =*t*_*s*_. TvSC, MZM, IDW and CDWI separately represent the trivial superconducting states, Majorana zero modes, incommensurate charge-density-wave liquids and commensurate charge-density-wave insulators. Phase boundaries between TvSC and MZM, MZM and IDW, TvSC and IDW, and IDW and CDWI, are described by black squares (corresponding to critical interaction ${U}_{1}^{c}$), red circles (${U}_{2}^{c}$), green stars (${U}_{{\rm{SL}}}^{c}$), and blue triangles (${U}_{{\rm{CDW}}}^{c}$), respectively.

The Hamiltonian Eq. (2) is effectively an 1D fermion chain. To study the many-body physics, we implement the DMRG method to perform numerical simulation, and investigate the ground state phase diagram. We compute the energy gap defined as the difference of the ground state energy in the even parity (*P* = 1) and odd parity (*P* = −1) sectors ${\rm{\Delta }}E=|{E}_{0}(P=+1)-{E}_{0}(P=-1)|$, the difference in paired entanglement spectra *δε* (*δε* = 0 indicates two-fold degeneracy of entanglement spectrum), charge structure factor *S*(*q*) (which indicate the strength of charge density wave with momentum *q*) and filling $\overline{n}$ of fermions (see **Method: Physical Quantities for the details**). There are several distinct phases such as trivial superconducting phase (TvSC), topological Majorana zero mode (MZM), incommensurate charge-density-wave liquid (IDW) and commensurate charge-density-wave insulators (CDWI) depending on the system parameters. We summarize the phase diagram as a function of *t*_*d*_ and *U* and at a variety of *t*_1, 2_ in Fig. 2.

**Figure 3:**
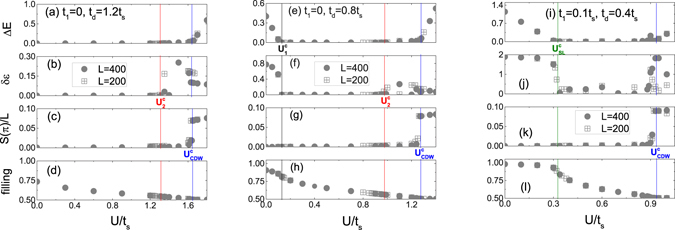
Key physical quantities vs *U*. From top to bottom rows: energy gap Δ*E*, quantity characterizing entanglement-spectrum degeneracy *δε*, charge structure factor at *q* = *π* (per length) *S*(*π*)/*L*, and filling of fermions $\overline{n}$, respectively. From left to right columns: (**a**–**d**) *t*_1,2_ = 0 and *t*_*d*_ = 1.2*t*_*s*_, (**e**–**h**) *t*_1,2_ = 0 and *t*_*d*_ = 0.8*t*_*s*_, and (**i**–**l**) *t*_1,2_ = 0.1*t*_*s*_ and *t*_*d*_ = 0.4*t*_*s*_, respectively.

First let us simply consider the *t*_1,2_ = 0 case as the chemical potential is adjusted at the surface Dirac node, i.e. the intra-leg hopping vanish, in Fig. 2(a) ^21^. In this case, the vortices on the same TI surfaces far separate in space. The noninteracting MZMs exist as *t*_*d*_ > *t*_*s*_. As the interaction is slightly turned on, we found that the ladder is still in the topological phase. Since the bulk-boundary correspondence still holds in interacting class D, a many-body MZM is localized on each end of the ladder for non-zero *U* ^23^. This many-body MZM is adiabatically connected to the single-particle MZM without the interaction. In Fig. 3(a–d), we show details of four physical quantities vs *U* at fixed *t*_*d*_ = 1.2*t*_*s*_ to identify the topological phase. The ground state energy in the even parity (*P* = +1) and odd parity (*P* = −1) sectors are doubly degenerate, so Δ*E* = 0 in Fig. 3(a). Furthermore, in Fig. 3(b) *δε* = 0, which indicates double degeneracy in the entanglement spectra, leads to the topological phase extended from *U* = 0. Upon increasing *U*, on the other hand, the smoothly decreasing filling $\overline{n}$ and the absence of featured peaks in *S*(*q*) show no indication of other physical phases.

**Figure 4:**
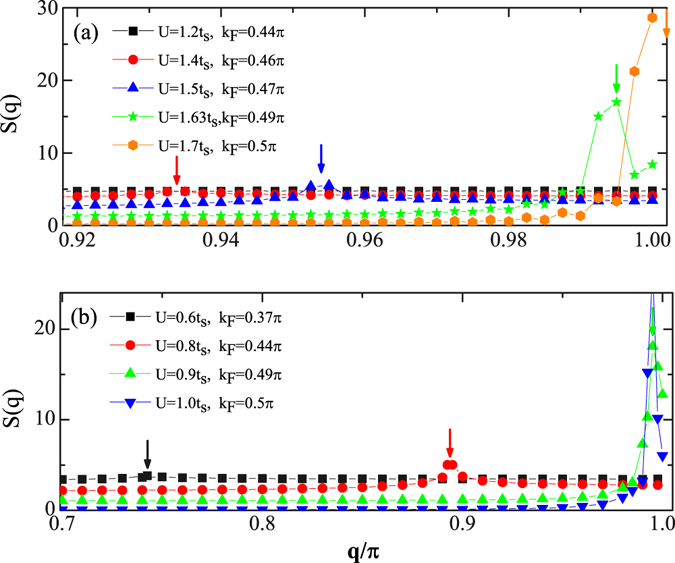
Charge structure factor *S*(*q*) of the Majorana ladder. The structure factor of the charge-charge correlation on the interacting Majorana ladder at (**a**) *t*_1,2_ = 0, *t*_*d*_ = 0.2*t*_*s*_ and (**b**) *t*_1,2_ = 001*t*_*s*_, *t*_*d*_ = 0.4 and various interaction strength (data are vertically shifted in arbitrary units for a clear view). The size is *L* = 400. The arrows point out the location of peaks.

The MZM phase region is extended as interaction *U* increases until the phase transition ${U}_{2}^{c}$ [red circles in Fig. 2(a)] to the incommensurate charge-density-wave liquid (IDW). To show the IDW region, we can see the double degeneracy between the even- and odd-parity ground states is clearly lifted^32^ (even if the energy gap is quite small) in Fig. 3(a). Meanwhile, as shown in Fig. 3(b) the double degeneracy of entanglement spectrum disappears, i.e. *δε* > 0. The charge structure factor *S*(*q*) shows peaks at the incommensurate wave vector at $q\cong 2{k}_{F}$, where *k*_*F*_ is the Fermi vector. An example can be seen Fig. 4(a) that the charge structure factors *S*(*q*) at different interaction strength as *t*_1,2_ = 0 and *t*_*d*_ = 1.2*t*_*s*_. In this regime, filling $\overline{n}$ still decreases smoothly upon increasing *U*, and the Fermi vector *k*_*F*_ as well as the peak locations of *S*(*q*) move towards to a larger *q*. This charge 2*k*_*F*_ instability of IDW state is also reminiscent of a similar feature of a Luttinger liquid^36, 37^. In Fig. 2(a), the red circles describe the phase boundary between MZM and IDW.

As *U* increases across the other phase boundary [blue triangles in Fig. 2(a) or blue line in Fig. 3(c)], the system opens a gap and a CDWI is detected. The dominant peak occurs at *q* = *π* and the CDW order parameter survives in the thermodynamic limit. At this moment, the filling approaches $\overline{n}\simeq 0.5$ or half-filling. The ground state is parity odd (*P* = −1) and Δ*E* ≠ 0. In a classical analogy, electrons are loaded on every other lattice site. The blue triangles (*U*_CDW_) depict the phase boundary between IDW and CDWI. By DMRG, we can distinguish the distinct phases and pin out the phase boundary by observing variations in Δ*E*, *δε* and *S*(*π*). The phase transition between IDW and CDW was also discovered theoretically by bond entropy method^38^. Experimentally, the appearance of CDWI can be measured by Coulomb drag^39^ or by thermodynamics method^40^.

Next we turn to the *t*_*d*_ < *t*_*s*_ regime, which physically corresponds to small tilted angle *θ* in Fig. 1(b). We used *t*_*d*_ = 0.8*t*_*s*_ as demonstration presented in Fig. 3(e–h). In the noninteracting limit (*U* = 0), the system is a trivial superconductor (TvSC) with a finite gap, because the Majorana hybridization *t*_*s*_ between the top and bottom TI surfaces destroys the topological phase. The ground state is parity even (*P* = 1) and the entanglement spectrum shows no paired degeneracy, so both ${\rm{\Delta }}E,\delta \varepsilon \ne 0$ at small *U* in Fig. 3(e,f). However, at a sufficiently strong (but not too strong) interaction strength, the ladder undergoes the topological phase transition at ${U}_{1}^{c}$, and MZMs emerge at each end of the ladder. The ground state has double degeneracy and the entanglement spectrum appears in pair, i.e. ${\rm{\Delta }}E=\delta \varepsilon =0$. Back to the phase diagram Fig. 2(a), we can clearly see that the topological state is adiabatically connected to the MZM in the *t*_*d*_ > *t*_*s*_ regime. This MZM is driven by finite interactions, as an interaction-induced topological state. The black squares in Fig. 2(a) describe the phase boundary between TvSC to MZM (our DMRG calculation shows a weak finite-size effect on the phase boundary). Upon increasing interaction strength, the MZM region is enlarged, implying that a moderate interaction stabilizes the topological MZM, even with less tilted magnetic fields. In the large-*U* side, the ground states are still characterized as the IDW and CDWI, similar to the observation in the *t*_*d*_ > *t*_*s*_ regime.

Next we move to consider ${t}_{1,2}\ne 0$ as the chemical potential is not located at the surface Dirac node. In reality, TI materials with chemical potential exactly at the Dirac node have not been discovered, so the intra-leg tunneling between the MZMs is inevitable. Therefore it is important to investigate how MZM responds to finite *t*_1,2_. The phase diagrams in Fig. 2(b) and (c) consider finite values of *t*_1,2_. The influence of *t*_1,2_ is remarkable in the interacting Majorana ladder. In Fig. 2(b) using *t*_1,2_ = 0.1*t*_*s*_, it is obvious to see that, compared to (a), where *t*_1,2_ = 0, the MZM regime shrinks. However, this phase still extends to a finite range whereas the IDW regime is enlarged. There exists a critical *t*_*d*_ to harbor the interaction-driven MZM, which is ${t}_{d}^{c}\sim 0.5{t}_{s}$. Below this point, the TvSC phase directly turns to the IDW state, and the MZM disappears. Both TvSC and IDW show trivial behavior in the entanglement spectra. To pin out the boundary, indicated by green stars in Fig. 2(b), we examine the gap magnitudes and observe *S*(*q*). At TvSC, ${\rm{\Delta }}E\ne 0$ and no featured peak in *S*(*q*), whereas at IDW, ${\rm{\Delta }}E\simeq 0$ and *S*(*q*) has a peak located at *q* = 2*k*_*F*_, as shown in Fig. 4(b). We summarize the variation of the physics observables at *t*_*d*_ = 0.4*t*_*s*_ and *t*_1,2_ = 0.1*t*_*s*_ and at variety of *U* in Fig. 3(i–l). At *U* = 0, there is an energy gap. The gap Δ*E* decreases to a small but finite value as *U* increases to ${U}_{SL}^{c}$ and remains small in ${U}_{SL}^{c}\le U\le {U}_{CDW}^{c}$. At $U > {U}_{CDW}^{c}$, Δ*E* rapidly increases and *S*(*π*) jumps to a finite value. In the whole range $\delta \varepsilon \ne 0$, so no MZM exists.

For further stronger intra-leg tunneling, the region of many-body MZMs becomes even smaller. Figure 2(c) shows the case of ${t}_{\mathrm{1,2}}=0.3{t}_{s}$. The critical *t*_*d*_ is estimated at ${t}_{d}^{c}\sim 0.8{t}_{s}$. Therefore, the presence of intra-leg tunneling *t*_1, 2_ will corrupt the stability of the many-body MZM. We have numerically estimated that, as ${t}_{1}={t}_{2}\,\gtrapprox \,0.5{t}_{s}$, the system no longer supports the interaction-driven MZM. This implies that the chemical potential has to be tuned to close to the surface Dirac node to suppress the intra-leg tunnelings. In experiment, the magnitudes of magnetic fields are required to be appropriately tuned, such that the vortices are away from each other to lower the intra-leg hybridization but close enough to strengthen the four-Majorana interaction.

## Discussion

With proper strength of the Majorana interaction, the topological region is tremendously enlarged; by tilting a small angle of magnetic field MZMs appear on the ladder ends, even in the presence of the Majorana hybridization between the top and bottom surfaces. As shown in Fig. 2 the intra-leg tunneling *t*_1,2_ of Majorana Fermions on the same surface shrinks the topological region. To enlarge the region, *t*_1,2_ can be tuned to zero by adjusting the chemical potential right at the surface Dirac node. For the recent experiment of the heterostructure on Bi_2_Se_3_ thin films^15^, we estimate the hybridization strength ${t}_{d,s}\sim 2$ meV and the interaction strength *U* ~ 0.56 meV so the ratio $U/{t}_{s}\sim 0.28$ (see **Method: Estimation of Majorana coupling and interaction**). Hence, as shown in Fig. 2(a), we can simply tilt the magnetic field such that ${t}_{d}/{t}_{s} > 0.6$ to expect the MZM on the end of vortex array of the naked surface. Our current proposal directly solves one of the major difficulties of the Fu-Kane model: usually the TI film has to be thin enough to induce the superconductivity gap on the naked surface, but such a thin film can lead to the Majorana hybridization, which destroys MZMs. Tilting the magnetic fields can both reduce the hybridization and enhance the interaction and hence rescue MZMs.

To explore other many-body phases, such as IDW and CDWI, one needs other TI materials to provide larger values of *U*/*t*_*s*_. It is interesting to see the transition between IDW and MZM, which only occurs with Majorana interactions. Such a topological phase transition is beyond the single-particle picture. The IDW state sharing similarities with a Luttinger liquid could be identified using the Coulomb drag measurement^39^.

Although the physics of many-body MZM and its topology has been discussed extensively^41–46^, promising platforms for such systems are barely found in the literature. In this report, we have designed a realizable experimental setup to investigate interaction effects on topological states.

## Method

### Noninteracting Majorana ladder

To determine the topological phase, we solve the ladder Hamiltonian ${\widehat{H}}_{{\rm{M}}}$ (1) as the interaction is off (*U* = 0) in the periodic boundary condition by extending the first summation to *L* and letting site *L* = 1 coincide with site 1. By performing Fourier transformation ${\gamma }_{j}=\frac{1}{\sqrt{L}}{\sum }_{k}{\gamma }_{k}{e}^{ijk},{\lambda }_{j}=\frac{1}{\sqrt{L}}{\sum }_{k}{\lambda }_{k}{e}^{ijk}$, the noninteracting Majorana Hamiltonian in momentum basis is given by

3 $${\hat{H}}_{{\rm{non}}}=\sum _{k}\frac{i}{2}(\begin{array}{cc}{\gamma }_{k}^{\dagger } & {\lambda }_{k}^{\dagger }\end{array})(\begin{array}{ll}2i{t}_{1}\,\sin \,k & {t}_{s}+{t}_{d}{e}^{ik}\\ -{t}_{s}-{t}_{d}{e}^{-ik} & 2i{t}_{2}\,\sin \,k\end{array})(\begin{array}{l}{\gamma }_{k}\\ {\lambda }_{k}\end{array}).$$

The topology of the Majorana ladder can be characterized by the Pfaffian of the Hamiltonian at *k* = 0 and *π*

$${(-\mathrm{1)}}^{\nu }={\rm{sgn}}({\rm{pfB}}\mathrm{(0)}{\rm{pfB}}(\pi ))={\rm{sgn}}(({{\rm{t}}}_{{\rm{s}}}+{{\rm{t}}}_{{\rm{d}}})({{\rm{t}}}_{{\rm{s}}}-{{\rm{t}}}_{{\rm{d}}})),$$

where $B(k)={t}_{s}+{t}_{d}{e}^{ik}$. Hence, the topological region is located at $|{t}_{d}| > |{t}_{s}|$ in this non-interacting system, irrespective of values of *t*_1_ and *t*_2_. We also expect *t*_1_ and *t*_2_ small enough to keep the system insulating. In the topological phase, the MZMs reside in the vortex cores whereas they vanish in the trivial phase as the magnetic field goes through the TI without tilting. By changing the tilted angle of the magnetic fields, one can manipulate the ratio of *t*_*d*_/*t*_*s*_ to trigger a topological transition between trivial and topological phases.

### Computational Methods

With a finite interaction in Eq. (2), exact characterization of the ground state is beyond the single-particle picture. Although one can still perform the Hartree-Fock approximation to decouple the interaction term as

$$\begin{array}{rcl}{n}_{j}{n}_{j+1} & \sim  & \langle {n}_{j}\rangle {n}_{j+1}+{n}_{j}\langle {n}_{j+1}\rangle -\langle {n}_{j}\rangle \langle {n}_{j+1}\rangle \\  &  & -({\chi }_{j}{c}_{j+1}^{\dagger }{c}_{j}+{c}_{j}^{\dagger }{c}_{j+1}{\chi }_{j}^{\ast }-|{\chi }_{j}{|}^{2}),\end{array}$$

with ${\chi }_{j}=\langle {c}_{j}^{\dagger }{c}_{j+1}\rangle$, the mean-field approach neglects the quantum fluctuations and may not accurately capture the ground state properties in one dimension. To study the many-body physics, we implement the density matrix renormalization groups method (DMRG) to the Hamiltonian of Eq. (2). The DMRG method has been shown to be an efficient numerical algorithm to describe one-dimensional correlated systems^24–27^ and has also been successfully applied on interacting systems with Majorana fermions^32, 33, 47, 48^. The approximated ground-state wave function as well as the entanglement spectrum can be easily obtained via iterative numerical renormalization. We set the number of states kept per block up to *m* = 120 and compare three different sizes *L* = 200, 400 and 600 to examine finite-size effects. Furthermore, we keep the truncation errors less than 10^−8^. Although the particle number is not conserved here, in the DMRG calculations, we can still employ parity $P={(-1)}^{{\sum }_{j}{n}_{j}}$ as a good quantum number to label the quantum state. Thus, the ground state energies *E*_0_(*P*) and the wave functions $|{{\psi }}_{0}(P)\rangle$ associated with the specific parity sector *P* are accessible.

### Physical Quantities

The first signature we use to identify the MZMs is a zero energy gap between the lowest even-parity and odd-parity states, ${\rm{\Delta }}E\equiv |{E}_{0}(P=1)-{E}_{0}(P=-1)|=0$. It reflects Majorana modes occupying two zero-energy levels, also causing double degeneracy in the ground state. The IDW phase has non-zero but small Δ*E* ^32^, while the other trivial phases have relatively large Δ*E*. Another signature to characterize the topological property is to compute the entanglement spectrum. The entanglement spectra {*ε*} are simply the eigenvalues of reduced density matrices

$${\rho }_{l}={{\rm{Tr}}}_{{r}}|{\psi }_{0}\rangle \langle {\psi }_{0}|,$$

where the subscript *l* represents partially tracing out the degrees of freedom of the right block. The topological phase has two-fold degeneracies of the entire entanglement spectrum. Rather than observing the entanglement spectrum, throughout the main context, we compute the unitless *δε* defined as

4 $$\delta \varepsilon =\sum _{P=\pm 1}\sum _{n}{({\varepsilon }_{n}^{P}-{\varepsilon }_{n+1}^{P})}^{2},$$

to distinguish the topological from trivial phases^49^. The first summation is over the ground states in two parity sectors. In the topological phase, both the ground state and the entanglement spectra are doubly degenerate, so all the paired entanglement spectrum difference $({\varepsilon }_{n}^{P}-{\varepsilon }_{n+1}^{P})$ vanish and *δε* = 0. This property is robust even in the presence of interaction^33^ and easily implemented with numerical simulation.

In the large-*U* limit, the system is a commensurate charge-density-wave (CDW) insulator, which is topologically trivial since neither the entanglement spectrum nor the ground-state energy shows double degeneracy. A CDW state has electrons residing on every other lattice sites (in a classical picture) to lower the interaction energy $4U{n}_{j}{n}_{j+1}$, and can hence be characterized by the structure factor of charge-charge correlations

5 $$S(q)=\frac{1}{L}\sum _{j^{\prime} ,j}\langle {n}_{j^{\prime} }{n}_{j}\rangle {e}^{iq({x}_{j^{\prime} }-{x}_{j})}\mathrm{.}$$

The CDW order parameter can be defined as ${O}_{{\rm{CDW}}}={\mathrm{lim}}_{L\to \infty }\sqrt{S(q)/L}$. In the thermodynamic limit, a finite *O*_CDW_ implies the existence of the long-range CDW ordering. At *q* = *π*, the CDW ordering is commensurate, labeled as CDWI in the phase diagrams in Fig. 2. For other values of *q*, it is incommensurate; in the main context, we have *q* = 2*k*_*F*_ instability in the incommensurate charge-density-wave liquid state, where *k*_*F*_ is the Fermi wave vector. In the spinless chain, half-filling $\overline{n}=0.5$ corresponds to ${k}_{F}=\pi \mathrm{/2}$. For $0.5 < \overline{n} < 1$, ${k}_{F}=(1-\overline{n})\pi$.

Figure 4(a) and (b) show the (unnormalized) charge structure factor *S*(*q*) for (a) *t*_1,2_ = 0, *t*_*d*_ = 1.2*t*_*s*_ (b) *t*_1,2_ = 0.1*t*_*s*_, *t*_*d*_ = 0.4*t*_*s*_. In both figures, at relative weak interaction strength, labeled by the black squares, no peaks are observed. They are located in the MZM and TvSC states in (a) and (b), respectively. At moderate interaction, *S*(*q*) develops peaks located roughly at *q* = 2*k*_*F*_. Upon increasing *U*, the filling $\overline{n}$ decreases and approaches to 0.5, and *k*_*F*_ moves toward to *π*/2. This feature reveals the 2*k*_*F*_ charge instability and characterizes the IDW state. At *q* = *π*, for (a) *U* = 1.7*t*_*s*_ and for (b) *U* = *t*_*s*_, the ground state is a CDWI, and consistently, $\overline{n}\simeq 0.5$ at this moment.

### Estimation of Majorana coupling and interaction

To estimate the strengths of the physical parameters, we consider the thin film of topological insulator Bi_2_Se_3_ on the top of the superconductor NbSe_2_, which is an experimental realization^15^ of the Fu-Kane model. First, the strengths of *t*_*s*_ and *t*_*d*_ stemming from the coupling of the top and bottom TI surface states are given by

6 $${t}_{s},{t}_{d}\sim {G}_{{\rm{bulk}}}{e}^{-{h}_{{\rm{TI}}}/{\lambda }_{{\rm{TI}}}}\sim 2{\rm{meV}},$$

where the bulk gap *G*_bulk_ of Bi_2_Se_3_ is about 0.3 eV. The thickness of TI on the superconductor in the recent experiment is 5 quintuple layers^14^, which is about *h*_TI_~5 nm. The decay length in the vertical direction is given by the Fermi velocity (*v*_*F*_ = 2.2 eV.*Å*) divided by the bulk gap $\hslash {\nu }_{F}/{G}_{{\rm{bulk}}}={\lambda }_{{\rm{TI}}}\sim 1$ nm^50^. This hybridization leads to non-zero energy Majorana fermions residing on the vortices.

We can estimate the values of *t*_1_ and *t*_2_ based on the parameters of the superconductivity, since in the absence of the superconductivity the Majoranas are delocalized on the top and bottom layers, regardless of superconductor proximity effect^20^, we use the NbSe_2_ superconducting gap *G*_SC_ ~ 1 meV. The estimated values of the intra-leg tunnelings are given by

7 $${t}_{1},{t}_{2}\sim {G}_{{\rm{SC}}}{e}^{-{d}_{{\rm{v}}}/{\lambda }_{M}}\sim 0.3\,{\rm{meV}},$$

where the distance between two Majoranas *d*_v_ on the same surface is about 50 nm and the decay length of Majorana hybridization strength on the topological insulator *λ*_*M*_ is close to the London penetration depth of the superconductor (40 nm) when the depth is smaller than $\hslash {\nu }_{F}/{G}_{{\rm{SC}}}$ ^28^. By comparing with *t*_*d*_ and *t*_*s*_, *t*_1_ and *t*_2_ can be neglected.

The interaction *U* for two Majoranas on the top and two Majoranas on the bottom comes from the Coulomb interaction of two electrons (holes), each of which is the overlap between two Majorana wavefunctions; hence, the strength of the interaction *U* of four Majorana *α*_1,2,3,4_ has been written in the density function *ρ* of electron (hole)^21^

8 $$U=-\mathrm{8(}{g}_{1234}+{g}_{4123}-{g}_{1234}),$$

where

$${g}_{ijkl}=\frac{1}{2}\int \int d{r}^{2}dr{^{\prime} }^{2}{\rho }_{ij}(r)V(r-r^{\prime} ){\rho }_{kl}(r^{\prime} ),$$

where *V*(*r* − *r*′) indicates the effective Coulomb potential. Since the overlap (${e}^{-{h}_{{\rm{TI}}}/{\lambda }_{{\rm{TI}}}}$) between the top and bottom TI surface Majoranas is less than on the same surface (${e}^{-{d}_{{\rm{v}}}/{\lambda }_{M}}$), the overlap of Majoranas on the surface is considered as major contribution to the interaction, or $\rho \sim {e}^{-{d}_{{\rm{v}}}/{\lambda }_{M}}$. The reason is that ${h}_{{\rm{TI}}}/{\lambda }_{{\rm{TI}}} > {d}_{{\rm{v}}}/{\lambda }_{M}$, The Coulomb potential, which can be estimated by the ionization energy of hydrogen *E*_*H*_, is given by $V=\frac{{E}_{{\rm{H}}}}{\varepsilon }\frac{{a}_{H}}{{h}_{{\rm{TI}}}}$, where *a*_*H*_ is the Bohr radius and the dielectric constant *ε* is about 20 due the screening of the Coulomb interaction. The value of the effective interaction energy is roughly

9 $$U\sim {\rho }^{2}V=\frac{{E}_{{\rm{H}}}}{\varepsilon }\frac{{a}_{H}}{{h}_{{\rm{TI}}}}{e}^{-2{d}_{{\rm{v}}}/{\lambda }_{M}}\sim 0.56\,{\rm{meV}}\mathrm{.}$$

This estimation is in agreement with ref. 21 which adopted another method for the estimation. Therefore, comparing the strengths of the interaction and hopping, we obtain the ratio *U*/*t*_*s*_ ~ 0.28.
